# Evaluating the Utility of the Surprise Question Among General Physicians for Appropriate Palliative Care Indication in Brazil

**DOI:** 10.1089/pmr.2024.0003

**Published:** 2024-07-19

**Authors:** Gabriel Barros Furtado Leão Borges, Cristiane Bitencourt Dias

**Affiliations:** São Paulo State Hospital for Civil Servants, São Paulo, Brazil.

**Keywords:** chronic diseases, palliative care, patient care planning, prognosis, SPICT, surprise question

## Abstract

**Objectives:**

This study aimed to assess the agreement between established tools, such as the Palliative Performance Scale (PPS) and Brazilian version of the Supportive and Palliative Care Indicators Tool (SPICT-BR), and the subjective assessment of palliative care (PC) need using the Surprise Question (SQ) administered by resident physicians. This assessment was conducted among hospitalized patients, with and without cancer, to determine the efficacy of these tools in indicating the need for PC.

**Methods:**

A six-month cross-sectional study in 2019 of medical records of patients hospitalized in a single center in IAMSPE-Brazil. The SPICT-BR and PPS were applied to the medical record data, and the SQ was posed to each resident physician. Comparisons for categorical data were made using the chi-square test, with *p* < 0.05 considered statistically significant.

**Results:**

Of 203 patients evaluated, 57.6% were male and 81.2% were older adults (≥60 years). The mean age was 67.40 ± 9.72 years. Chronic disease was nonneoplastic in 78.32% of patients, and 56.65% had not been hospitalized in the preceding year. The PPS score was <70% in 69.4% of patients, and 51.2% met at least one SPICT-BR criterion. Among patients with cancer, 40.9% had over two positive SPICT-BR criteria; 97.5% of these patients received NO responses to SQ by residents (*p* < 0.0001). Similarly, 90.6% of patients with one SPICT-BR criterion received NO responses to SQ, with no significant difference between groups.

**Conclusion:**

The SQ proved to be a valuable tool for PC indication, particularly when administered by untrained professionals. Consistent with SPICT-BR findings, our study highlights the SQ’s role in facilitating early identification of patients in need of PC.

## Introduction

Understanding the importance of improving palliative care (PC) diagnosis in Brazil is paramount, especially given the current landscape characterized by a scarcity of studies, limited inclusion of PC in medical curricula,^1^ and insufficient knowledge about PC among health care professionals.^2^ Early identification of patients who would benefit from PC can significantly improve their quality of life and reduce unnecessary suffering. In Brazil, where access to specialized PC services is often limited, early diagnosis becomes even more critical, as it allows for timely implementation of appropriate interventions and support measures.^3^

Various tools have emerged to facilitate the identification of patients with chronic illness who might benefit from PC, with the goal of enhancing their quality of life,^4,5^ though many of them require experience or training to be applied. In this way, to have an early identification of patients who need this specific care, the intuitive question or Surprise Question (SQ) was created, which is posed to the physician:^5–9^ “Would you be surprised if this patient were to die in the next 12 months?” which shows an accuracy of 52.9%^5^ for the need of PC compared to two-year mortality assessment. The SQ has been shown to be efficient in identifying patients at higher risk of death among those with chronic kidney disease undergoing hemodialysis and among those with cancer.^8,9^ It has been used in emergency, primary, and inpatient care.^9–11^ Although, some papers show that untrained physicians might lack accuracy when answering the SQ.^12^ SQ sensitivity and specificity for identifying patients who may require PC were reported as 69% and 99%, respectively.^13^

In 2010, another prognostic tool—the Supportive and Palliative Care Indicators Tool (SPICT)—was created by the University of Edinburgh Palliative Care Research Group and the National Health Service Lothian.^14^ The SPICT has the objective of enabling the identification of patients who may benefit of PC and providing the necessary guidelines to structure care plans for such patients.^15^ It operates by flagging individuals with deteriorating health owing to one or more advanced conditions, prompting health care providers to consider PC interventions. The SPICT emphasizes early identification to optimize patient care and ensure appropriate support is provided throughout the illness trajectory. Data show that 48% of patients who meet one of the SPICT criteria will die in the next 12 months. In a study of 501 patients with ≥70 years of age, the SPICT was found to have a sensitivity of 81% and a specificity of 98%.^13^ Therefore, the SPICT offers a method that explains the general indicators of worsening health.^14^

Previous studies have demonstrated the practical utility of SPICT in identifying patients who could benefit from PC. In primary care settings, SPICT can aid in identifying patients who are suitable candidates for PC,^16^ and it is more likely to be positive in older, male patients with more comorbidities, having been shown to be a good predictor of one-year mortality,^17^ as well as to decrease the likelihood of unnecessary interventions.^18^

In oncology, one of the most commonly utilized performance scales is the Palliative Performance Scale (PPS),^19–22^ which functions as a prognostic tool specific for use in PC settings, for estimating the survival of patients with chronic diseases. It is observed that as physical performance declines, prognosis worsens.^10,22–27^ In addition, PPS facilitates prognosis estimation for patients with chronic progressive and incurable diseases^11^ and aids in identifying the natural progression of various illnesses.^26^ PPS is a widely used tool for assessing the functional status of patients with advanced illness. It evaluates performance in activities of daily living and mobility, providing a numerical score ranging from 0% (bedridden) to 100% (fully functional), and values <70% indicate reduced functionality.^22^

The PPS aids in prognostication and informs clinical decision-making by quantifying functional decline and identifying patients who may benefit from PC services, which has also been validated for use in Brazil.^28^ Scores ranging from 10% to 30% are associated with different survival periods, typically between 1 and 30 days. In addition, PPS scores <70% have shown improved predictive accuracy within a 90-day timeframe.^29^ Some studies have employed two distinct strata: one with scores ≥70%, linked to higher functionality and life expectancy, and another with scores <70%, associated with increased one-year mortality risk.^30^

By integrating PC principles into clinical practice early in the disease trajectory, health care professionals can better address patients’ physical, psychosocial, and spiritual needs, leading to improved patient outcomes and enhanced patient and family satisfaction. Moreover, improving PC diagnosis has the potential to alleviate the burden on health care resources by minimizing unnecessary hospitalizations, aggressive treatments, and futile interventions. Therefore, fostering awareness and improving PC diagnosis in Brazil is essential to ensure that patients receive optimal care aligned with their values, preferences, and goals throughout the course of their illness.^31^

There is still a gap in our understanding of how resident physicians subjectively evaluate the need for PC compared to established tools. Some studies suggest potential benefits of utilizing resident physicians assessments, such as the SQ. However, further research is needed to fully elucidate the efficacy and implications of this approach in clinical practice.^32^

Hence, this study aims to evaluate the concordance between established tools including the PPS and Brazilian version of the Supportive and Palliative Care Indicators Tool (SPICT-BR) applied by a senior physician, compared to the one-year clinical assessment of PC need through the SQ applied by resident physicians. Through this investigation, we seek to promote the integration of subjective assessments into clinical practice, potentially facilitating the early identification and initiation of PC interventions for patients in need by untrained professionals.

## Methods

### Study description

**Fig. 1.**
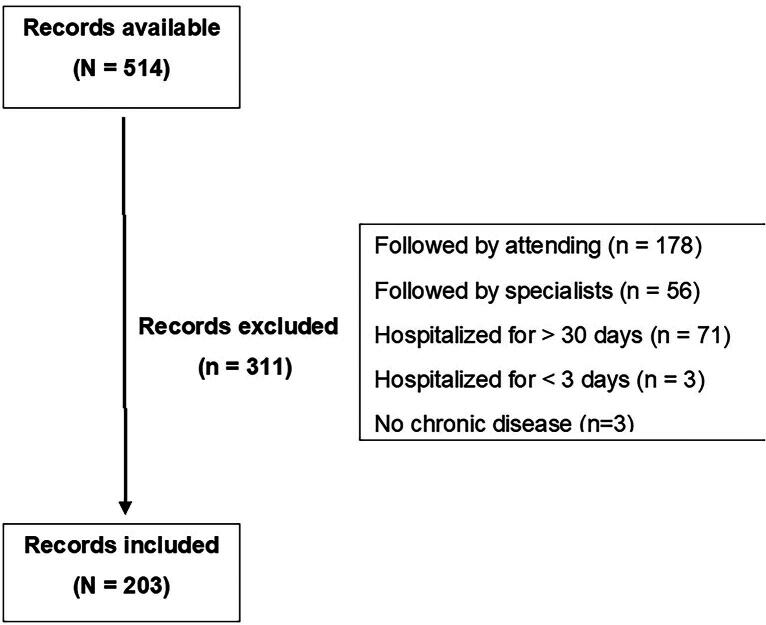
Flow chart of the selection process.

This study was a cross-sectional analysis conducted over six consecutive months in 2019, utilizing electronic medical records of patients admitted to the Internal Medicine ward of the São Paulo State Hospital for Civil Servants, located in São Paulo, Brazil (Fig. 1). The inclusion criteria comprised inpatients of age ≥18 years, hospitalized for 3–30 days with a chronic disease, and under the care of internal medicine residents. The 30-day window was chosen because the data were collected monthly retroactively, ensuring that the same patient would not be accounted for more than once. Based on their age at admission, patients were categorized as either young (<60 years of age) or older adults (≥60 years of age). The data were stratified by age because we have distinct populations admitted to the hospital: young individuals with preserved functionality and low morbidity burden compared with older adults with compromised physical performance and more severe diseases. The author applied both the SPICT-BR and the PPS using the information available in the medical records. Both the PPS and SPICT are validated instruments in Brazil, with the latter being applicable by identifying any clinical indicators of one or multiple life-limiting conditions. In this study, a resident physician is defined as an individual who has completed their medical degree within the past two years and is still in their first year of residency specialization training. Each resident physician in internal medicine who followed the patients was posed with the SQ.

### Data collection

The author collected data on several variables including age, gender, race, education level, income, length of hospital stay, underlying disease or diagnosis of chronic disease, and hospitalization history. In addition, the author performed the PPS score and determined the number of SPICT-BR criteria met by each patient. Following patient evaluation, the SQ was posed to the first-year resident physician on the day of the patient’s admission to the internal medicine infirmary.

A YES response to the SQ indicated that death within the next year would be surprising, whereas a NO response indicated that death within the next year would not be surprising. The residents were not provided with training on how to answer the question or what the answer would imply, thus allowing for a more subjective result. The staff coordinating the residents, including internal medicine experts, geriatricians, cardiologists, nephrologists, rheumatologists, gastroenterologists, and pneumologists, were not involved in the questioning and did not influence the residents’ responses.

Chronic disease was defined as any condition present in the SPICT chart, regardless of whether it scored positive or not for PC. Patients with missing information were excluded from the study. We selected a PPS cutoff of 70, as it indicates functional impairment, thus serving as evidence of disease. In addition, in many medical records, it was not feasible to accurately determine patients’ functionality owing to insufficient detailed information, with only indications of reduced functionality being available. In addition, publications have shown that PPS demonstrates higher accuracy in patients below that cutoff.^33^

### Statistical analysis

Numerical data were presented as mean ± standard deviation or median with interquartile range when appropriate and categorical data by absolute number and percentage. Comparison between two groups of numerical data was performed using the paired *t*-test or Wilcoxon test, as appropriate, whereas categorical data were compared using the chi-square test, with the level of significance set at *p* < 0.05. A pilot study conducted in the medical ward, where the proportion of PC indication was 59% (p1) versus 41% (p2) without indication, utilized the proportion study formula to calculate the sample size for the study. With a power of 80% and an alpha error of 5%, the power coefficient was 7.9, which yielded a sample size of 118 patients in both the palliative and nonpalliative groups. All statistical tests were performed with GraphPad Prism software, version 9.1 (GraphPad Software Inc., San Diego, CA, USA).

### Ethical aspects

The study was approved by the Research Ethics Committee of the São Paulo State Hospital for Civil Servants (Reference no. 981). Because all data contained in the medical records analyzed were treated confidentially, the requirement for informed consent was waived.

To ensure confidentiality with research information, all data are securely stored in a password-protected electronic database accessible only to authorized personnel. Access to sensitive information is restricted to individuals directly involved in the research project, and they were trained on the importance of maintaining confidentiality. When presenting or sharing research findings, personally identifiable information were anonymized to prevent identification of individual participants. In addition, all researchers adhered to relevant ethical guidelines and regulations regarding data protection and confidentiality, such as obtaining informed consent from participants and complying with institutional review board requirements.

## Results

We assessed a total of 514 medical records of patients hospitalized between May and October 2019. Of these, 311 (60.5%) were excluded: 234 due to lack of follow-up by internal medicine residents, 74 due to hospitalization duration <3 days or >30 days, and 3 due to absence of chronic disease. Thus, the study sample comprised 203 patient records.

Table 1 displays the demographic and clinical characteristics of the sample. Among the 203 patients evaluated, 117 (57.6%) were male and 165 (81.2%) were older adults, with a mean age of 67.40 ± 9.72 years. In total, 159 patients (78.3%) had nonneoplastic diseases, among whom 83 (40.8%) had cardiovascular disease. Furthermore, 115 patients (56.6%) had not been hospitalized in the past year and 44 (21.6%) had cancer. There was no statistically significant difference between patients with and without cancer regarding mean age (65.41 ± 9.72 vs. 67.96 ± 9.68 years, *p* = 0.123), mean number of hospital admissions in the past year (0.61 ± 0.57 vs. 0.44 ± 0.59, *p* = 0.097), or mean PPS score (47.95 ± 22.26 vs. 55.03 ± 20.8, *p* = 0.050).

**Table 1.** General Characteristics of the Sample

Characteristic	(n = 203)
Age (years), mean ± SD	67.40 ± 9.72
≥60 (*n* = 165)	71.08 ± 5.85
18–59 (*n* = 38)	51.47 ± 6.87
Sex, *n* (%)	
Male	117 (57.6)
Female	86 (42.4)
Type of chronic disease, *n* (%)	
Non-neoplastic	159 (78.3)
Cardiovascular	83 (40.8)
Neoplastic	44 (31.6)
Hospitalization in the past year, *n* (%)	
Yes	88 (43.3)
No	115 (56.6)
PPS score, *n* (%)	
≥70%	62 (30.5)
<70%	141 (69.4)
SPICT-BR criteria met, *n* (%)	
One or more	104 (51.2)
None	99 (48.7)

PPS, Palliative Performance Scale; SD, standard deviation; SPICT-BR, Brazilian version of the Supportive and Palliative Care Indicators Tool.

Of the 203 patients in our sample, 141 (69.4%) had a PPS score <70%, and 104 (51.2%) met at least one SPICT-BR criterion. However, the mean number of SPICT-BR criteria met was significantly higher among patients with cancer (1.47 ± 1.04 vs. 0.60 ± 0.81, *p* < 0.001). Among the 141 patients with a PPS score <70%, the resident physician answered NO to the SQ in 100 (70.9%) cases, compared with 41 (29%) for patients with a PPS score ≥70%, and this difference was statistically significant (*p* < 0.0001). In addition, the SPICT-BR identified one or more positive criteria in 104 patients. Among them, 99 (95.2%) exhibited a PPS score <70% (*p* < 0.0001). Conversely, among the 99 patients with a negative SPICT-BR, 42 (42.5%) had a PPS score <70%.

In addition, 18 (40.9%) of the patients with cancer had more than two positive SPICT-BR criteria, whereas this occurred in only 22 (13.8%) of the remaining 159 patients (*p* < 0.0001). Moreover, among the 40 patients with two or more SPICT-BR criteria, the residents responded with NO to the SQ for 39 (97.5%) of them (*p* < 0.0001). Conversely, out of the 64 patients with one SPICT-BR criteria, the residents answered NO to the SQ 58 (90.6%) times, with no statistically significant difference observed between one or two positive criteria SPICT-BR groups.

On older adults, the mean age was 71.08 ± 5.85 years, and the mean PPS score was 52.12% ± 21.63%. In comparison, among younger patients (Table 2), the mean age was 51.47 ± 6.87 years, and the mean PPS score was 59.47% ± 18.74%. There was no statistically significant difference between the two age groups regarding the mean PPS score (*p* = 0.0731). Of the 165 older adults, 77 (46.66%) met none of the SPICT-BR criteria, 56 (33.94%) met one criterion, and 32 (19.40%) met two or more criteria. Similarly, of the 38 younger patients, 22 (57.90%) met none of the SPICT-BR criteria, 8 (21.05%) met one criterion, and 8 (21.05%) met two or more criteria. There was no statistical difference between the two age groups regarding the mean number of SPICT-BR criteria met (*p* = 0.3449).

**Table 2.** Comparison between Older Adults and Younger Patients

Variable	Older adults	Young	p
	*(*n* = 165)*	*(*n* = 38)*	
Age (years), mean ± SD	71.08 ± 5.85	51.47 ± 6.87	<0.001
PPS score (%), mean ± SD	52.12 ± 21.63	59.47 ± 18.74	0.0731
SPICT-BR criteria met, *n* (%)			
None	77 (46.6%)	22 (57.9%)	
One	56 (33.9%)	8 (21%)	0.3449
Two or more	32 (19.4%)	8 (21%)	

Table 3 presents the breakdown of resident physician responses to the SQ, stratified by SPICT-BR result. Among the 104 patients who met at least one SPICT-BR criterion, the resident physician answered YES to the SQ in only seven (6.7%) cases. In contrast, among the 99 patients who did not meet any of the SPICT-BR criteria, the resident physicians responded positively to the SQ in 87 (87.8%) cases (*p* < 0.0001). This indicates a statistically significant correlation between a positive response to the SQ (answering NO) and the presence of positive SPICT-BR criteria. Table 4 displays the breakdown of resident physician responses to the SQ stratified by underlying disease type. Among the 44 patients with cancer, the resident physicians answered NO to the SQ in 36 (81.8%) cases compared with 73 (45.9%) for the 159 patients without cancer (*p* < 0.0001).

**Table 3.** Relationship between the Surprise Question and the Brazilian Version of the Supportive and Palliative Care Indicators Tool

Response to the SQ	SPICT-BR criteria met		Total
	One or more	None	
YES (*n*)	7	87	94
NO (*n*)	97	12*	109
Total (*n*)	104	99	203

SQ, Surprise Question (“Would you be surprised if this patient were to die in the next 12 months?”).

^*^ *p* < 0.0001 meeting at least one SPICT-BR criterion versus meeting no criteria.

**Table 4.** Relationship between the Surprise Question and the Type of Chronic Disease

Response to the SQ	Type of chronic disease		Total
	Neoplastic	Non-neoplastic	
YES (*n*/%)	8/18.2	86/54.1	94
NO (*n*/%)	36/81.8	73/45.9*	109
Total (*n*)	44	159	203

SQ, Surprise Question (“Would you be surprised if this patient were to die in the next 12 months?”).

^*^ *p* < 0.0001 patients with neoplastic disease versus those without neoplastic disease.

## Discussion

Our sample predominantly comprised hospitalized older adults with chronic nonneoplastic diseases. Almost 70% of the patients had a PPS score <70%, and roughly half met at least one SPICT-BR criterion. We found no statistically significant difference between older adults and younger patients regarding PPS scores or the number of SPICT-BR criteria met, contrary to our initial expectations given the higher morbidity burden typically observed in older populations.

The SQ was administered to resident physicians responsible for each patient, and the results were compared with the number of SPICT-BR criteria met and PPS scores. Proportionally, meeting at least one SPICT-BR criterion corresponded to a negative response to the SQ. The subjective perception of the resident physicians correlated well with predictors of advanced disease, signs of functional decline, and progression to the end of life, as evaluated by the SPICT-BR.

One study demonstrated that the SQ exhibits a sensitivity of 69.3% and a specificity of 83.7% in identifying patients with cancer who require PC.^34^ In our study, we observed a higher proportion of physicians responding NO to the SQ for patients with cancer compared with those without. These findings suggest that physicians may find it more straightforward to consider palliative and end-of-life care for patients with malignancies than for those with advanced chronic nonneoplastic diseases. Despite this, we found that the subjective assessment by resident physicians using the SQ correlated with the indication for PC and demonstrated agreement when compared to a more comprehensive tool, such as SPICT-BR, irrespective of the type of disease.

The 2010 SPICT does not establish a minimum value to indicate PC, as did previous versions (e.g., ≥2 criteria in its 2014 version). However, studies conducted in Japan and Belgium have suggested that there is a need to use a minimum number of SPICT criteria.^16^ Our study did not find a statistically significant relationship between the number of positive SPICT-BR criteria likely due to the limited number of participants. In addition, it is noteworthy that the only patient with more than two SPICT-BR criteria who received a NO response to the SQ was a 29-year-old woman with hematological malignancy. Hematological malignancies are recognized for delays in PC recommendations, which may have influenced this result.^35^

Given that PC is symptom-centered, an increase in positive SPICT-BR criteria indicates a greater need for specialized care from a PC specialist. In Brazil, where there is a shortage of referral centers, it is crucial to adopt a pragmatic approach for patient follow-up. In the authors’ opinion, refractory symptoms and PC/oncology emergencies should be prioritized. However, patients with a higher disease impact, as reflected by more positive SPICT-BR criteria, should be given priority alongside these emergencies.

Despite finding no difference in PPS scores between patients with and without cancer in our sample, a greater proportion of patients with cancer met at least one SPICT-BR criterion. For patients with a neoplastic disease to meet a positive SPICT criterion, they typically demonstrate functional decline or are deemed ineligible for treatment. In our study, our infirmary exclusively admitted patients who either did not qualify for antineoplastic therapy or were hospitalized for other reasons. Given the characteristics of our sample, individuals with cancer also presented with a higher burden of comorbidities, reduced performance status, and potentially influencing the answers given by resident physicians. Nonetheless, some studies have demonstrated^36^ that implementing SPICT increases the likelihood of identifying patients with nonneoplastic disease who require PC, despite the persistent notion that PC is exclusively for patients with cancer or terminal disease.^37^

In our study, patients with a PPS score <70% (indicative of lower functionality) had a higher estimated one-year mortality rate, as determined by the SQ. Interestingly, resident physicians reported that death would not be surprising in nearly 30% of patients with a PPS score ≥70% (indicative of higher functionality). This suggests that PC should not be reserved solely for end-of-life or low-functionality patients.

The limitations of this study are that the data were obtained solely from medical records, and physical examinations by the author were not conducted, potentially impacting the accuracy of the applied assessment tools. Although medical residents received training in the application of the PPS and were instructed to document relevant information, the lack of direct examination may have influenced the completeness and accuracy of the data. Moreover, the awareness among residents regarding the research’s focus on PC may have introduced bias in their responses to the SQ. Furthermore, the absence of follow-up assessments to evaluate intervention outcomes restricted the comprehensive understanding of the study’s impact. All physicians involved in this study were internal medicine residents without specific training in PC. Therefore, we cannot generalize our findings to physicians in other residency programs or specialists. Lastly, the inability to precisely stratify the degree of functional loss using the PPS through medical record review posed a challenge in accurately assessing patients’ functional status.

Given that our study population mainly comprised older adults with nonneoplastic diseases, reflecting the hospital’s epidemiological profile and selection bias toward patients with severe, refractory diseases, our findings may not be generalizable. However, the distribution of neoplastic and nonneoplastic diseases aligns with general population expectations.^26^ The proportion of patients for whom PC may be warranted based on SPICT results was consistent with existing literature.^38^

The findings of this study underscore the importance of adopting a patient-centered approach to improve the identification of patients requiring PC. The insights gained from this research have significant implications for clinical practice, particularly in settings where PC remains underdeveloped or inadequately addressed.

## Conclusion

Our study highlights the utility of the SQ as a valuable tool for PC indication, particularly when administered by untrained professionals. Our findings, consistent with those obtained using the SPICT-BR, emphasize the SQ’s role in facilitating early identification of patients in need of PC. Despite its subjectivity, the SQ proves effective in prompting further evaluation and investigation into PC needs, complementing more comprehensive assessment tools. By integrating the SQ into routine clinical practice, health care providers can better identify and address the PC needs of patients with chronic diseases, ultimately leading to improved patient outcomes and reduced health care costs.

## Abbreviations Used

IAMSPE: São Paulo State Hospital for Civil Servants
PC: Palliative care
PPS: Palliative Performance Scale
SPICT-BR: Supportive and Palliative Care Indicators Tool
SQ: Surprise Question
